# Vitamin D deficiency, bone turnover markers and causative factors among adolescents: a cross-sectional study

**DOI:** 10.1186/s40200-016-0266-2

**Published:** 2016-10-12

**Authors:** Bagher Larijani, Arash Hossein-Nezhad, Elham Feizabad, Zhila Maghbooli, Hossein Adibi, Majid Ramezani, Eghbal Taheri

**Affiliations:** Osteoporosis Research Center, Endocrinology and Metabolism Clinical Sciences Institute, Tehran University of Medical Sciences, 5th floor, shariati Hospital, North Kargar, Tehran, 141142386 Iran

**Keywords:** Vitamin D deficiency, Bone turnover markers, Adolescents

## Abstract

**Background:**

This cross-sectional population-based study was conducted to elucidate the prevalence of vitamin D deficiency, bone turnover marker’s variation and its influencing factors among adolescents of Tehran.

**Methods:**

Totally 444 middle and high school (53.6 % in high school) students (both girls and boys) were recruited. A short food frequency questionnaire designed to estimate dietary calcium and vitamin D consumption. Serum levels of calcium, phosphorus, parathyroid hormone (PTH), bone specific alkaline phosphates, 25 (OH) vitamin D, osteocalcin, cross-linked C-telopeptide (CTX), total protein, albumin and creatinine were determined.

**Results:**

Vitamin D deficiency was prevalent in adolescents and only 22.4 % of students had normal serum vitamin D. Results revealed that vitamin D insufficiency reported in 34.2 % of students and vitamin D deficiency was in 43.3 % of them. Serum vitamin D, osteocalcin, CTX and bone specific alkaline phosphates were significantly higher in boys in all different ages. Serum levels of 25 (OH) vitamin D had positive influences on bone turnover markers and had negative correlation with PTH.

**Conclusions:**

Vitamin D deficiency and insufficiency is common among healthy adolescents of Tehran. There is a pressing need to improve vitamin D status among adolescents. Increasing vitamin D fortification of dairy products can be considered as a population-wide public health strategy in Iran.

## Background

As evidences increasingly emphasis that 25 (OH) vitamin D deficiency, in addition to its negative impact on bone mineral density status, may contribute to other chronic disease, such as cardiovascular, hypertension, diabetes, cancer, increaces incidence of schizophrenia and depression and some inflammatory and autoimmune diseases, to fight 25 (OH) vitamin D deficiency in all age groups is a very important measure [1–3].

The prevalence of 25 (OH) vitamin D deficiency in adolescents is different, but considerably high in many regions, like Middle-east and South-East Asia. High prevalence of 25 (OH) vitamin D deficiency in Iran is similar to the results of other studies in Middle East areas, 75.1 % of women and 72.1 % of men in Iran have different level of 25 (OH) vitamin D deficiency [4, 5].

In addition to high prevalence of 25 (OH) vitamin D deficiency in Iranian adult, 25 (OH) vitamin D deficiency in Iranian adolescents is common for an example, prevalence of 25 (OH) vitamin D deficiency in Tehran adolescent is 53.6 % in girls and 11.3 % in boys. 25 (OH) vitamin D deficiency in female students was about five times more common than male students [6–8].

In children and adolescents 25 (OH) vitamin D deficiency is more important because development of peak bone mass happens in these critical ages and any adverse effects due to low 25 (OH) vitamin D and low calcium level will influence in their later lifelong [9, 10]. For example, one of the factors that can decrease the chance of osteoporosis in old ages is peak bone mass during childhood and adolescence [11].

Also, 25 (OH) vitamin D deficiency can have adverse effects on bone health. It is well known that prolonged 25 (OH) vitamin D deficiency (serum 25 (OH) vitamin D concentrations <10–25 nmol/L) can cause rickets in children and osteomalacia in adults. Although, the influence of subclinical 25 (OH) vitamin D deficiency or 25 (OH) vitamin D insufficiency on skeletal health is less obvious [12].

It is well-established that, in elderly people, low 25 (OH) vitamin D status increases parathyroid hormone (PTH) concentrations in serum, that causes bone turnover and bone loss, defects mineralization, and increases risk of fractures of bones [13]. But the effect of increasing PTH in children and adolescents is unclear [12]. For example, serum PTH concentration is normally higher during adolescence [14, 15].

Bone formation marker, Osteocalcin and a new marker of bone resorption, carboxyl terminal telopeptide of type I collagen (CTX) [11] can help us to understand more about what happen during 25 (OH) vitamin D deficiency in adolescents.

According to the above-mentioned facts and low available evidences, the present study was done to assess the level of serum 25 (OH) vitamin D and bone turnover markers and other contributing bone health in adolescents lived in Tehran.

## Method

The cross-sectional study is conducted on guidance and high school students of both genders from different districts of Tehran, the Iranian capital, in wintertime to assess the level of serum 25 (OH) vitamin D and bone turnover markers and other contributing bone health.

From 36 schools of 5 different district of Tehran, Three middle schools and three high schools for boys were randomly selected from the list. A similar strategy was used to select the girl schools. The selected schools were distributed in five different regions of Tehran Municipality including 3, 10, 11, 16, and 17. Students studying in region three included 27.3 % of participants, region ten 8.3 %, region eleven 25.9 %, region sixteen 20 %, region seventeen 18.5 %.

This research was carried out in compliance with the Helsinki Declaration and approved by ethics committee, Institute of Endocrinology and Metabolism, Tehran University of Medical Sciences. All the parents sign an informed consent.

Inclusion criteria included all subjects that educated in these selected schools and their parents signed informed consent form and the students themselves tend to participate in our study. Exclusion criteria included students who have taken vitamin D supplements (different forms of Calcium and Vitamin D supplements or multivitamins containing Calcium and Vitamin D) during the past 3 months, suffering from underlying conditions (liver, kidney, gastrointestinal, cancer, endocrine, bone and biliary disease) or consuming medication affecting bone metabolism (anti-convulsants, anti-tuberculosis medication, HMG-CoA inhibitors, cimetidine, theophylline, and cholestyramine), as well as those who are following special diets such as vegetarian diet or consuming fortified products regularly.

Approximately 10 cc of blood was drawn between 7:00 A. M. and 9:30 A. M. after the student had fasted for at least eight hours. Blood samples were used to determine calcium, phosphorus, bone specific alkaline phosphatase, 25 (OH) D, osteocalcin, cross-linked C-telopeptide (CTX), total protein, albumin and creatinine. Blood samples were taken in a sitting position according to the standard protocol. Participants rested in the seated position for 15 min prior to blood collection. This waiting period allowed calibration of the concentrations of blood components. The vacuum tubes were immediately placed on wet ice and transferred to the EMRI laboratory for centrifugation (at 3000 rpm for 10 min).

Blood samples were aliquoted into four vials for freezing at -70 °C until assayed. Calcium (Man, Arsenazo), phosphorus (Man, Phosphomolybdate, UV), albumin (Pars Azmoon, Bromocresol Green), total protein (Pars Azmoon, Biuret), creatinine (Man, Jaffe Kinetic), and alkaline phosphatase (Pars Azmoon, DGKC) were determined with inter assay coefficient variation (CV) of 3.9, 6, 2.2, 3, 4.5 and 2.1 % respectively. Intact PTH was measured by Immunoradio Metric Assay (IMMUNOTECH) with CV of 6.6 %. Vitamin D, Osteocalcin (N- MID), CTX (Immunodiagnostic systems) were measured by Enzyme Immunoassay with CV% of 9, 8, 6 and 10 % respectively. All the samples with high concentration of analytes (more than reportable ranges) were diluted according to kit protocol and assessed again. The normal ranges of blood vitamin D for adolescents considered >30 ng/ml. Serum 25-hydroxyvitamin 20 ≤ D ≤ 30 ng/ml considered as vitamin D Insufficiency and serum 25-hydroxy vitamin D <20 ng/ml as vitamin D deficiency. The normal ranges of blood calcium for adolescents considered 6.7-10.7 mmol/L.

The students are then asked to fill a questionnaire on their demographic data, possible underlying diseases and bone health-related lifestyle habits. They also asked to fill a weekly questionnaire on their compliance with milk consumption and possible reasons for not consuming milk regularly. Also, a short food frequency questionnaire designed to estimate dietary calcium and vitamin D consumption in the past 3 months.

Finally, the data were analyzed with SPSS version 19. *T*-test, ANOVA and Spearman correlations tests were used. We use parametric tests because the numbers of students in each group of analyses were more than thirty. *P* value less than 0.05 was considered to be statistically significant.

## Result

The total number of participations were 444 people and the number of girls and boys were approximately equal (48.2 vs. 51.8 %). The average age of them was 14.34 and about 46.4 % of them were studying in middle school and 53.6 % in high school.

The average calcium intake in boys’ daily diet was 1240.2 mg (±72.45) while this was 1108.05 mg (±62.34) in girls but about 40 % in each sex groups consumed calcium below 700 mg per day. The mean daily vitamin D intake in boys was 1.42 microgram (±0.08) and in girls was 1.29 microgram (±0.08).

The average of serum calcium was 9.93 mg/dl (±0.02) and the minimum of calcium level was 8.10 mg/dl and it showed that no students had below normal calcium level whereas only 22.4 % of them had normal ranges of serum 25 (OH) vitamin D.

**Fig. 1:**
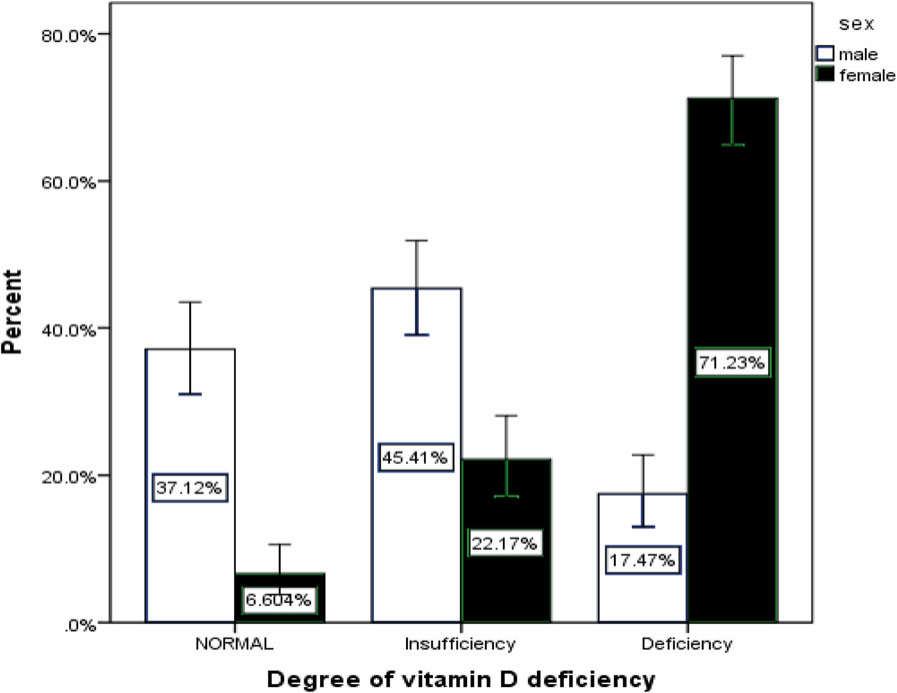
The prevalence of 25 (OH) vitamin D deficiency in different genders. 25 (OH) vitamin D normal range: serum 25 (OH) D >30 ng/ml. 25 (OH) vitamin D Insufficiency: serum 25 (OH) D 20 ≤ D ≤ 30 ng/ml. 25 (OH) vitamin D Insufficiency: serum 25 (OH) D 20 ≤ D ≤ 30 ng/ml

The mean of serum 25 (OH) vitamin D was 25.82 ng/ml (±0.74) and the minimum of 25 (OH) D was 4.37 ng/dl. About 34.2 % of students had 25 (OH) vitamin D Insufficiency (serum 25-hydroxyvitamin 20 ≤ D ≤ 30 ng/ml) and 43.3 % 25 (OH) vitamin D deficiency (serum 25-hydroxy vitamin D < 20 ng/ml). Also, the prevalence of 25 (OH) vitamin D deficiency was showed separately according to different genders in Fig. 1.

The level of 25(OH) D was significantly higher among boys rather than girls, 31.32 ng/ml vs 19.89 ng/ml while the level of serum calcium higher in girls.

We assessed bone mass markers in boys and girls separately in age ≤ 14 years and age > 14 years (because this age separates middle and high school students and also, most girls come in puberty stages before the age of 14, while most boys come in puberty stages after this age). Serum 25 (OH) vitamin D, osteocalcin, CTX and bone specific alkaline phosphates were significantly higher in boys in two categories of ages and Parathyroid hormone had a significant different between boys and girls but with higher level in girls (Table 1).

**Table 1:** The Mean ± SE of bone markers and serum biomarkers according by age, sex

	Boys ≤ 14YO	Girls ≤ 14YO		Boys > 14YO	Girls < 14YO	
	*N* = 124	*N* = 105	*P*-value (*t*-test)	*N* = 106	*N* = 109	*P*-value (*t*-test)
Calcium (mg/dL)	9.91 ± 0.05	10.00 ± 0.05	0.216	9.81 + 0.05	9.99 + 0.04	0.013
Phosphorus (mg/dL)	5.03 ± 0.05	4.44 ± 0.06	<0.001	4.48 ± 0.06	4.06 ± 0.05	<0.001
25 (OH) D (ng/mL)	33.94 ± 1.38	19.65 ± 1.64	<0.001	28.27 + 1.30	20.12 + 1.12	<0.001
Parathyroid hormone (pg/mL)	50.29 ± 3.46	92.66 ± 9.45	<0.001	36.18 + 2.01	43.29 + 2.40	0.025
Osteocalcin (ng/mL)	91.02 ± 3.29	59.93 ± 2.04	<0.001	65.99 + 3.23	25.67 + 1.17	<0.001
CTX (pg/mL)	1.76 ± 0.06	1.01 ± 0.05	<0.001	1.45 + 0.06	0.49 + 0.02	<0.001
Bone specific alkaline phosphates (mcg/L)	251.57 + 9.59	174.17 + 12.76	<0.001	138.59 + 9.23	43.81 + 1.28	<0.001
Albumin (g/dL)	5.38 ± 0.02	5.24 ± 0.02	0.001	5.24 ± 0.03	5.17 ± 0.02	0.095
Total protein (g/dL)	8.30 ± 0.04	7.72 ± 0.05	<0.001	8.23 ± 0.04	7.76 ± 0.04	<0.001
Creatinine (mg/dL)	0.80 ± 0.01	0.75 ± 0.01	0.002	0.92 ± 0.01	0.88 ± 0.008	0.051

*SE* standard error of mean, *YO* years old, *CTX* cross-linked C-telopeptide

**Fig. 2:**
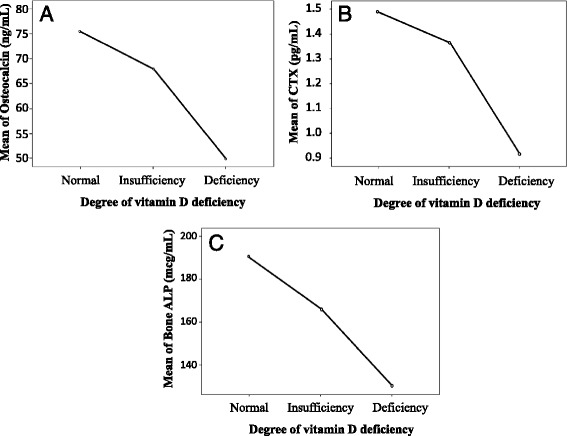
Osteocalcin (**a**), CTX (**b**), Bone alkaline phosphatase (**c**) trends in different serum 25 (OH) vitamin D categories.25 (OH) vitamin D normal range: serum 25 (OH) D >30 ng/ml, 25 (OH) vitamin D Insufficiency: serum 25 (OH) D 20 ≤ D ≤ 30 ng/ml, 25 (OH) Vitamin D deficiency: serum 25 (OH) D < 20 ng/ml. CTX: cross-linked C-telopeptide. Bone ALP: Bone alkaline phosphatase

A negative significant correlation between serum 25 (OH) vitamin D and PTH was detected. Also, there was a significant positive correlation between serum 25 (OH) vitamin D and osteocalcin, CTX, bone specific alkaline phosphates (Fig. 2). As was shown in Fig. 2, with increasing in serum 25 (OH) vitamin D, bone turnover markers increased. Also, the mean of each bone markers, can be significantly altered based on changing in serum 25 (OH) vitamin D categories that was shown in Table 2.

**Table 2:** *P*-value measurement (ANOVA Post Hoc test*) between bone markers level and 25 (OH) vitamin D different categories

Bone markers	*P*-value between bone markers level and 25 (OH) vitamin D different categories(ANOVA Post Hoc test)		
	Normal range with Insufficiency	Normal range with deficiency	Deficiency with Insufficiency
Osteocalcin	0.291	<0.001	<0.001
CTX	0.42	<0.001	<0.001
Bone alkaline phosphatase	0.31	<0.001	0.03

*: As number of students in each of groups of vitamin D deficiency were more than 30 and Test of Homogeneity of Variances was nonsignificant (>0.05) Scheffe Post Hoc was used, *p*-value was significant at <0.05

## Discussion

The present study showed that 25 (OH) vitamin D deficiency in Tehran healthy adolescents, was common and it was about 43.3 %, with higher rates in girls by 71.23 % rather than boys by 17.47 % and it was near to previous study that was assessed the prevalence of 25 (OH) vitamin D deficiency in Tehran adolescents. In that study, Prevalence of serum 25(OH) vitamin D <20 ng/ml was 53.6 % in girls and 11.3 % in boys [16].

Also, our study showed that in female adolescents, 25 (OH) vitamin D deficiency, was about four times more prevalent than males (*p* < 0.001), it was constant with previous reports and need to attention to this major health problem, not just for female adolescents, also for next generations [16, 17].

Different factors may cause 25 (OH) vitamin D deficiency in Tehran adolescents, as Middle East habitants, despite its abundant sunshine, they are more susceptible to25 (OH) vitamin D deficiency [18]. In addition to Iran, low serum 25(OH) D levels had been reported in the different region of Middle East, for example, Turkey, Lebanon and Jordan, also, in these countries serum 25 (OH) vitamin D was lower in women rather than in men. It is worth to mention that the immigrants in Europe that come from Middle East and Asia carry a high risk for 25 (OH) vitamin D deficiency [19].

Synthesis of 25 (OH) vitamin D in the skin is very important and it contains about 90 % of all the body’s needs [20], but many determinants can influence in the synthesis of 25 (OH) vitamin D in the skin including exposure to UVB solar radiation, winter season, sunscreen use, being indoors [21–23]. Tehran is a mega city with polluted air. Air pollution is one of the main elements influences in the percentage of the ground level UVB. The level of air pollution is negatively associated to the amount of solar UVB that reaches earth surface, as a result, more pollutant areas, less UVB passage and consequently, 25 (OH) vitamin D cutaneous syntheses reduces [24].

Natural dietary sources of 25 (OH) vitamin D are very few and foods that are fortified with 25 (OH) vitamin D are often inadequate to satisfy either a child’s or an adult’s 25 (OH) vitamin D requirement [3, 25].

On the other hand, adolescence is critical ages to skeletal growth and reach to optimal peak bone mass. The enough intake of calcium and 25 (OH) vitamin D from daily dietary, in addition to the normal serum range of calcium and 25 (OH) vitamin D, has positive effects on bone in adolescents. For example, milk consumption positively correlates with bone mineral density of the total body, spine and radius in adolescent girls [26–29].

Considering the above-mentioned facts, increasing vitamin D fortification of dairy products can be recommended as a population-wide public health strategy to fight 25 (OH) vitamin D deficiency especially in adolescents.

As it was shown in our study, there was a significant positive correlation between serum 25 (OH) vitamin D with osteocalcin, cross laps, bone specific alkaline phosphates. While Bone markers assessment and it’s associations is rare in other last studies.

In adult, increasing in serum 25 (OH) vitamin D suppresses serum PTH and decreasing serum 25 (OH) vitamin D increases serum PTH concentrations, but the effects of increasing in PTH in children and adolescents are unclear, although in our study, a negative significant correlation was detected between serum 25 (OH) vitamin D and PTH in adolescents [12, 1, 13].

One of things, that could confounded our result was, not excluding students with positive history of use any supplements consuming or any underlying diseases after sampling (9 students consumed different forms of Calcium and Vitamin D supplements, 1 person had jaundice in face and hands, 1 person kidney stones, 4 students gastrointestinal disorder, 5 students thyroid disorder and 1 person had rickets. one person had seizure disorder and consumed valproate sodium, one person used atorvastatin and two people consumed corticosteroid drugs), but we try to find these not correct reports of these students with double checking and minimize their confounded effects. Also, due to the small sample size, our study could not be representieveness of all Tehran adolescents, although, our study had an acceptable power in determining the prevalence of vitamin D deficiency.

The present study had some limitations. In this study, only adolescents living in the city have been studied. Further research might explore adolescents living in rural areas to determine the amount of Vitamin D and calcium deficiency and bone health biomarkers levels. Furthermore, the impact of this suboptimal vitamin D status on bone health in both population subgroups needs to be investigated. This study was more comprehensive than earlier research and collected data on PTH, and biomarkers of bone formation and resorption in relation to vitamin D status that were not previously evaluated. Hence, data obtained in the present study gives a comprehensive insight into the influence of vitamin D status and biochemical markers of bone turnover in Tehran’s adolescent, after taking into consideration other known factors such as Food habits, the amount of vitamin D intake and lifestyle-related factors.

The findings of the present study show that suboptimal vitamin D status and 25 (OH) vitamin D deficiency is very common in Tehran’s adolescents. It can have adverse effects on bone health, especially in these sensitive ages. Also, the results of this study indicate that 25 (OH) vitamin D deficiency in female adolescents was about four times more prevalent than males. It is becoming increasingly clear that the fundamental unit for nutrition is the food (eg, milk, nuts, eggs), not the nutrient (e.g., calcium, saturated fat, cholesterol). A nutrient perceived as beneficial, such as calcium, may be unhealthy if the parent food, say milk, contains other nutrients, such as galactose, that on the balance might stimulate adverse effects in the body. In theory, consuming calcium-rich foods such as bones, fermented dairy (e.g., unsweetened yogurt, kefir, and cheese), leafy greens, almonds, and chia seeds may be an effective strategy for improving both calcium intake and long-term health. With regard to results of this study, vitamin D supplementation was associated with the absence of severe deficiency and considerably lowered the prevalence of marginal deficiency of vitamin D in adolescents in the present study.

## Conclusions

Vitamin D deficiency and insufficiency is common among healthy adolescents of Tehran. There is a pressing need to improve vitamin D status among adolescents. Increasing vitamin D fortification of dairy products can be considered as a population-wide public health strategy in Iran.

## Abbreviation

25 (OH) D: 25 hydroxy vitamin D
CTX: Cross-linked C-telopeptide
